# A Rare Incidence of Metachronous Neurovascular Lesions in a Child

**DOI:** 10.7759/cureus.11270

**Published:** 2020-10-31

**Authors:** Deepak Chandrasekaran, Emmanuel D Azariah, Elengkumaran S, Ravindran Chinnaswami, Vijayanirmala Subramani

**Affiliations:** 1 Oral and Maxillofacial Surgery, Sri Ramachandra Institute of Higher Education and Research, Chennai, IND; 2 Oral and Maxillofacial Pathology, Sri Ramachandra Institute of Higher Education and Research, Chennai, IND

**Keywords:** mesenchymal tumor, solitary, intraosseous, juvenile, benign, hemimandibulectomy, spindle cells, immunohistochemistry, metachronous, trucut biopsy

## Abstract

Neurofibroma is an uncommon benign tumor arising from nerve sheath fibroblasts. The diagnosis of solitary lesions becomes difficult in patients who do not have any family history of neurofibroma. An intra-oral solitary neurofibroma comprises 6.5% of reported cases of neurofibroma. Few cases of intraosseous solitary neurofibroma have been published as per literature. Treatment-surgical resection of tumor has an excellent prognosis with extreme rare malignant transformation which is more commonly seen in neurofibromatosis. A periodic follow-up is necessary in solitary tumors to rule out syndromic cases. Leiomyoma is a benign tumor of smooth muscle origin, which is usually diagnosed in the gastrointestinal tract, uterus, and skin. The most effective treatment for solid, vascular, and epithelioid angioleiomyomas is surgical resection along with tumor capsule. Here we report an uncommon occurrence of multiple benign tumors in a pediatric patient.

## Introduction

The intraosseous solitary neurofibroma is rare occurrence of the oral cavity, with the most common site being the mandible [1]. There is a definite female predilection (2:1) and the mean age of occurrence is 27.5 years. Discomfort, pain, or paresthesia are common clinical manifestations of the intraosseous variant. Roentgenographically, it appears as a well-circumscribed or poorly defined radiolucent lesion [2]. The gross appearance of the tumor is not encapsulated and has a softer consistency. A scrupulous histopathological analysis shows numerous spindle cells with elongated or oval nuclei in a myxoid matrix [3]. The treatment of intraosseous neurofibroma is radical surgery which includes hemimandibulectomy or enbloc resection of the mandible [4]. In this case report, we report a patient diagnosed with neurofibroma based on biopsy; however afterward six postoperatively metachronous tumors were identified in the same site.

## Case presentation

**Figure 1:**
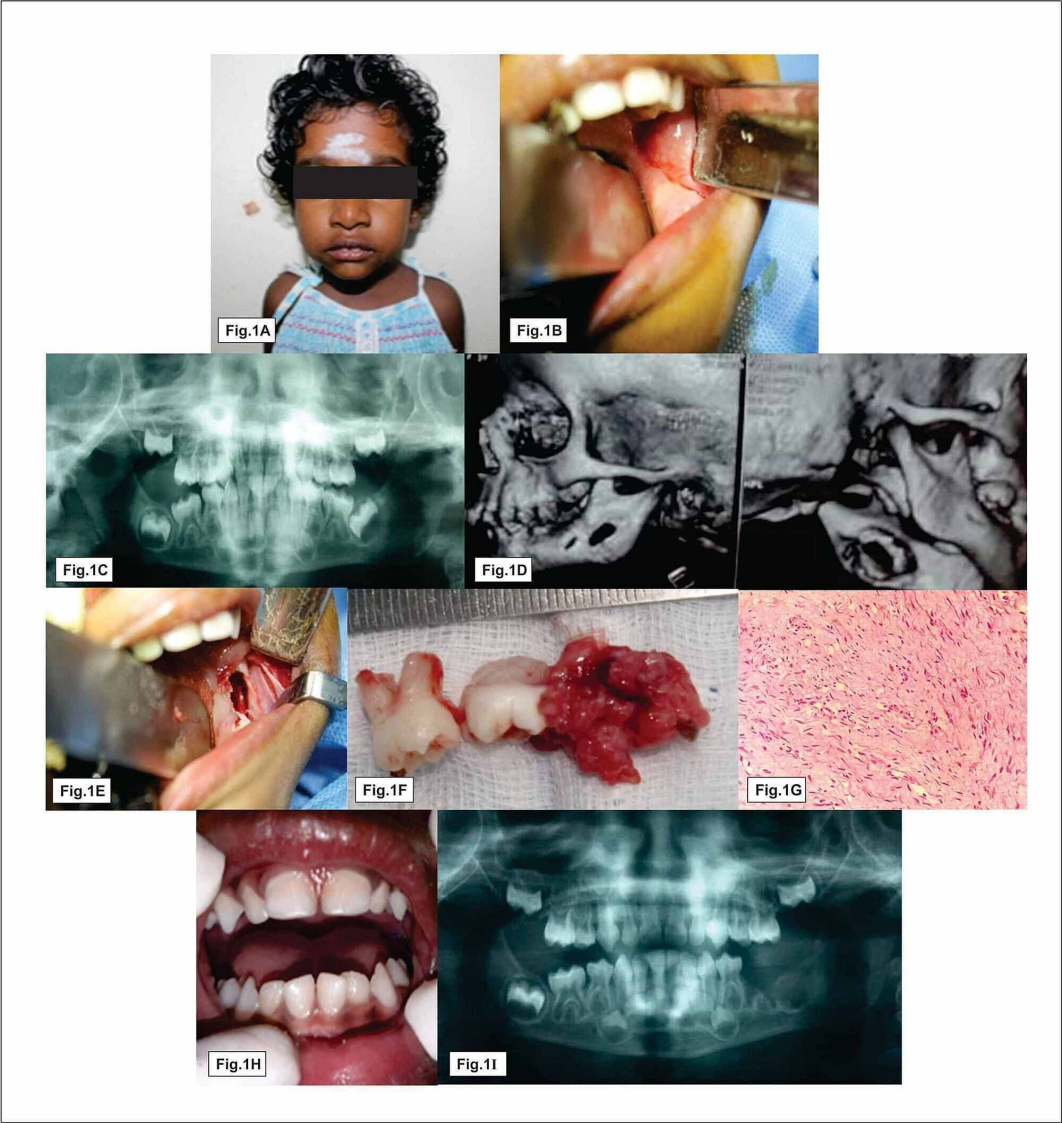
Pre- and post-operative clinical, radiographic and histopathological features of primary lesion of neurofibroma Fig. 1A & 1B show facial asymmetry and intraoral lesion, Fig. 1C & 1D represent radiolucency involving the body of the mandible, Fig. 1E & 1F show surgical excision and specimen, Fig. 1G shows spindle cell with wavy nuclei, Fig. 1H & 1I represent postoperative view

**Figure 2:**
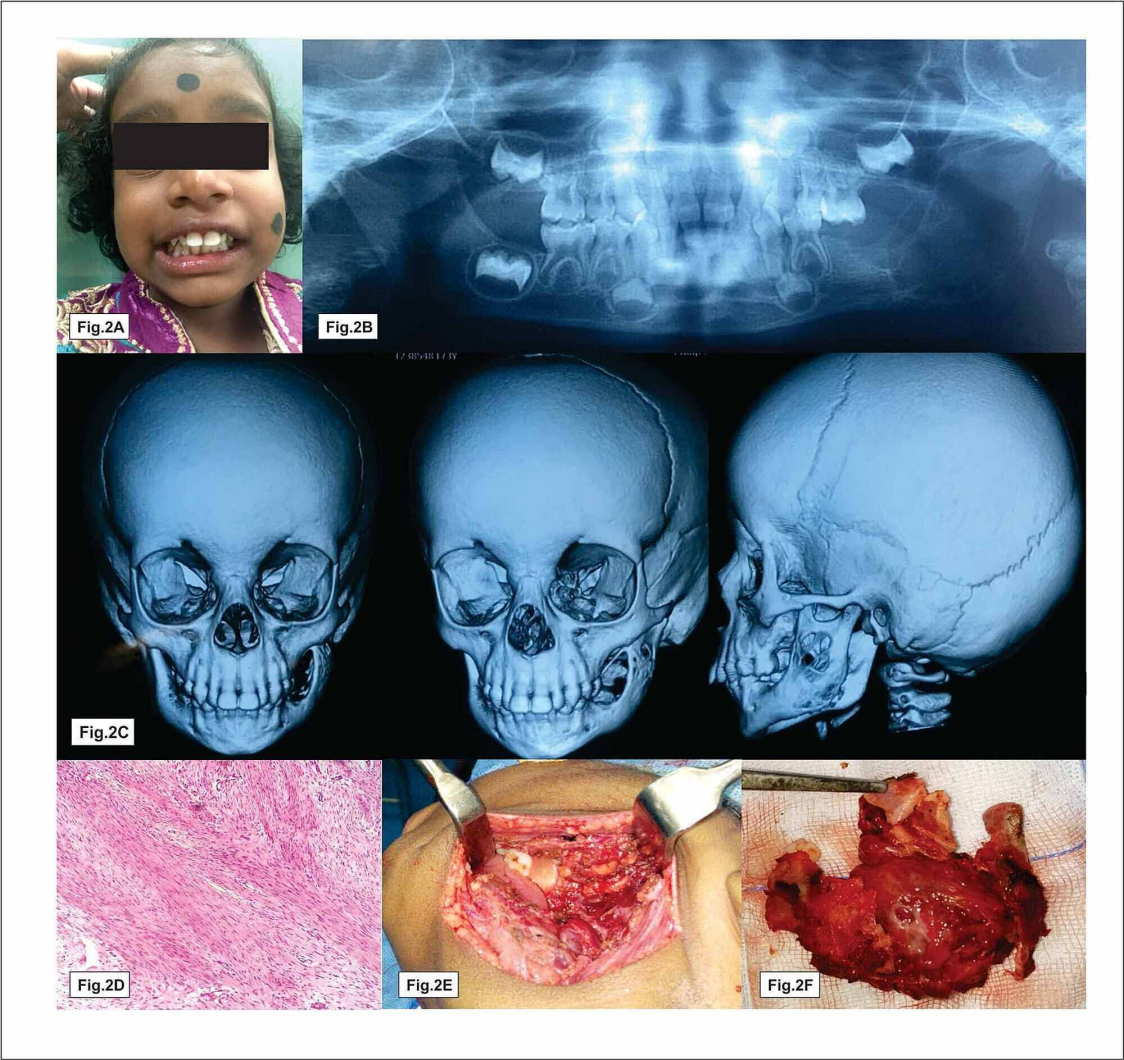
Clinical, imaging and histopathological features of metachronous lesion of angioleiomyomas Fig. 2A shows patient inability to open mouth, Fig. 2B & 2C represent osteolytic lesion involving the left ramus, Fig. 2D shows uniform spindle cell with blunt ended nuclei in fibrous connective stroma and also slit like vessels present, Fig. 2E & 2F show hemimandibulectomy and resected specimen

**Figure 3:**
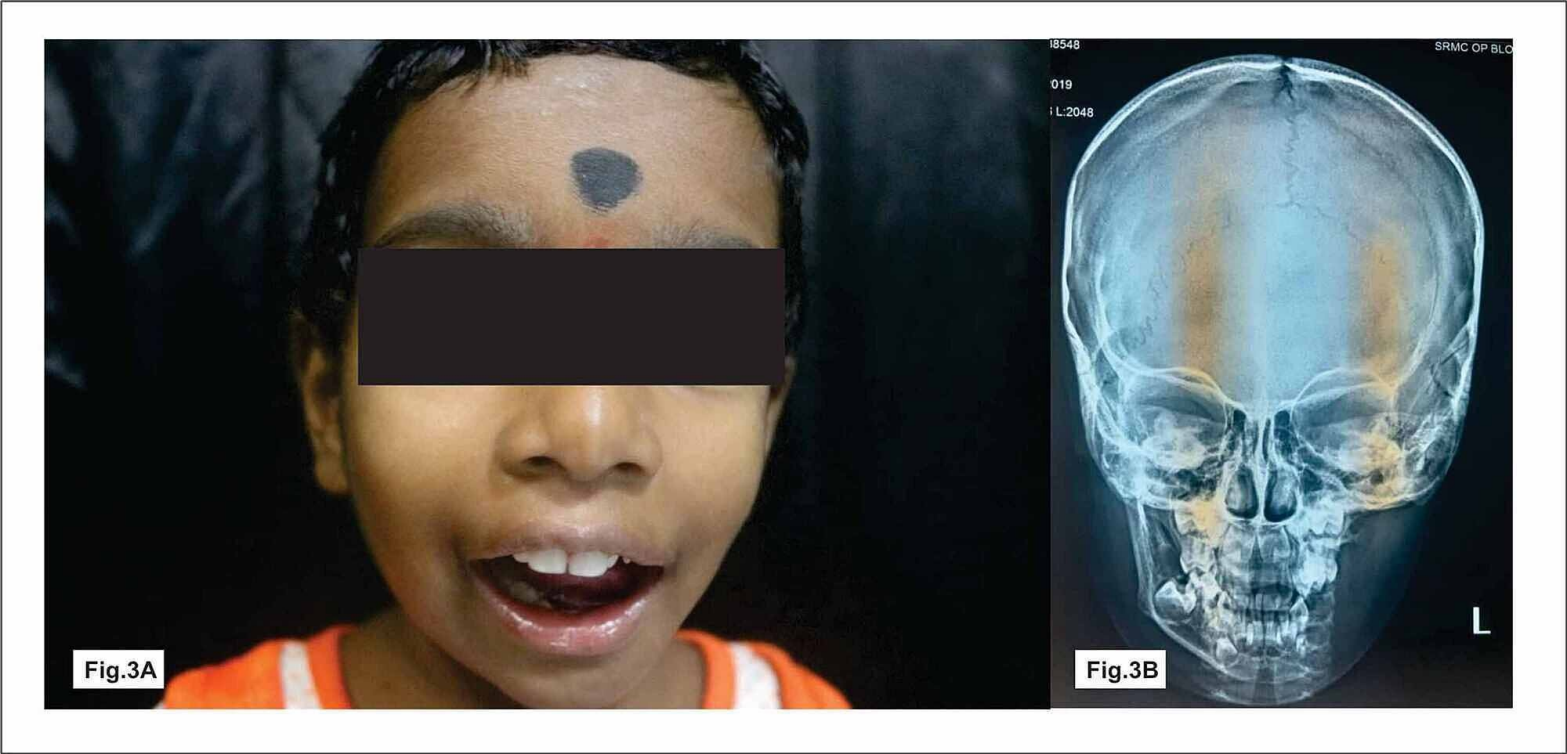
Postoperative follow-up view

A patient originally presented with a history of mild pain and inability to open the mouth for three months. Extraoral examination revealed mild swelling in relation to the left side of the face with a restricted mouth opening (Figure 1A). Intraoral swelling measured about 1.5 x 1cm, tender hard in consistency (Figure 1B). Orthopantomogram (OPG) and computed tomography (CT) images revealed an irregular osteolytic expansile lesion with soft tissue component measuring 1.8 x 1cm involving the ramus and angle of the mandible (Figure 1C, 1D). Hemogram showed elevated alkaline phosphatase level; other parameters were within normal limits. Based on these findings, we arrived at a provisional diagnosis of dentigerous cyst or eosinophilic granuloma. After informed written consent was obtained from the patient‘s parent, the patient underwent surgical excision under general anaesthesia (Figure 1E). A horizontal linear incision was made over the lesion. The lesion was localized and the excised specimen sent to histopathology. The pathology report reveals tumor areas composed of elongated spindle-shaped cells with wavy nuclei distributed in the form of interlacing bundles. A few areas of storiform appearance of bundles were also seen and inferred to be neurofibroma (Figure 1F). Postoperative review, the patient was pain-free with complete resolution of symptoms (Figure 1G, 1H). After six months of follow-up, the patient had same complaints in the same site (Figure 2A). OPG and three-dimensional facial scan revealed a well defined expansile soft tissue density with osteolytic lesion involving the ramus and angle of the left mandible and also involving submandibular fossa and masticator space (Figure 2B, 2C). Tru-Cut biopsy was performed and microscopic report revealed streaming fascicles of spindle-shaped cells with wavy nuclei in dense collagenous connective stroma (Figure 2D). Immunoprofiling showed vimentin-positive, S100, beta-catenin, smooth muscle actin, and cluster of differentiation 117 (CD117) were negative and Ki67 was less 1% positive, suggestive of atypical neurofibroma/fibromatosis. Based on aggressive clinical behavior, imaging features histopathological report with immunoprofiling, surgeons planned for left hemimandibulectomy under general anesthesia (Figure 2E, 2F). Excised specimen sections showed circumscribed lesion spindle cells arranged in fascicles and bundles. Adjacent fibromuscular tissue was seen. Vimentin was positive, smooth muscle actin (SMA)-focal positive, Ki67 less 7%, negative S100 which was in favor of benign vascular leiomyoma. Follow-up examinations at six months, one year, and 1.5 years showed no sign of recurrence clinically as well as radiologically (Figure 3A, 3B).

## Discussion

Intraosseous angioleiomyomas are extremely rare tumors, particularly in the head and neck. They predominantly affect males between the fourth to sixth decades [5]. In contrast, our patient was the youngest female have been reported, similar to Bertolini et al. [6]. Clinical manifestation of intraosseous leiomyoma varies from asymptomatic to non-specific pain with facial asymmetry. Radiographically, most of the cases were of unilocular radiolucency, but which may be multilocular with either ill- or well-defined sclerotic borders. Cortical destruction or expansion with root resorption or tooth displacement are seen depending on the nature and location of the lesion. The radiographic differential diagnosis of this lesion includes odontogenic origin lesions, central giant cell lesion, traumatic bone cyst, myxoma, and haemangioma [7]. The typical cigar-shaped nuclei arranged in whirls and interlacing bundles and the brighter eosinophilic cytoplasm in leiomyoma. Immunohistochemical stains with a panel of antibodies like S100, SMA, caldesmon, desmin, HHF-35, calponin, and Ki-67 helped to differentiate angioleiomyoma from other types of spindle cell tumors in special involving young individuals and to confirm the histopathological diagnosis. Therefore, immunohistochemistry plays a vital role in distinguishing spindle cell neoplasms. In the reviewed literature, wide surgical resection is the most successful with insignificant recurrence [8]. Metachronous is diagnosed six months after the surgery for the primary lesion and located in the same or different site. Incidence of metachronous benign tumors in the pediatric population are rarest [9] and the present report was one such case.

## Conclusions

Our case report of metachoronous lesion of neurofibroma and vascular angioleiomyoma is a poignant reminder that rigorous attention must be paid to the the clinical and radiological follow-up of these lesions and one must be cognizant of the possible malignant transformation. Follow-up aims at early diagnosis and treatment of metachronous lesions that can appear many years after diagnosis of the primary lesion.
